# Using whole genome scores to compare three clinical phenotyping methods in complex diseases

**DOI:** 10.1038/s41598-018-29634-w

**Published:** 2018-07-27

**Authors:** Wenyu Song, Hailiang Huang, Cheng-Zhong Zhang, David W. Bates, Adam Wright

**Affiliations:** 1 Division of General Internal Medicine and Primary Care, Brigham and Women’s Hospital and Harvard Medical School, Boston, Massachusetts 02120 USA; 2 Department of Biomedical Informatics, Harvard Medical School, Boston, Massachusetts 02115 USA; 3 Analytic and Translational Genetics Unit, Massachusetts General Hospital and Harvard Medical School, Boston, Massachusetts 02114 USA; 4 Department of Biostatistics and Computational Biology, Dana-Farber Cancer Institute and Harvard Medical School, Boston, Massachusetts 02215 USA; 5 Broad Institute of MIT and Harvard, Cambridge, Massachusetts 02142 USA; 6 Information Systems Department, Partners HealthCare, Somerville, Massachusetts 02145 USA

## Abstract

Genome-wide association studies depend on accurate ascertainment of patient phenotype. However, phenotyping is difficult, and it is often treated as an afterthought in these studies because of the expense involved. Electronic health records (EHRs) may provide higher fidelity phenotypes for genomic research than other sources such as administrative data. We used whole genome association models to evaluate different EHR and administrative data-based phenotyping methods in a cohort of 16,858 Caucasian subjects for type 1 diabetes mellitus, type 2 diabetes mellitus, coronary artery disease and breast cancer. For each disease, we trained and evaluated polygenic models using three different phenotype definitions: phenotypes derived from billing data, the clinical problem list, or a curated phenotyping algorithm. We observed that for these diseases, the curated phenotype outperformed the problem list, and the problem list outperformed administrative billing data. This suggests that using advanced EHR-derived phenotypes can further increase the power of genome-wide association studies.

## Introduction

A fundamental goal of precision medicine is to use genomic data to explain and predict health status. Studies have shown that many human complex disorders are driven by genomic factors^1–3^. In light of these findings, researchers are trying to further address the causal relationship between genetic variations and specific diseases phenotypes.

One challenge researchers facing regularly is the lack of accurate phenotype data which can match with genomic information^4^. In many cases, given the perfect genomic profiles generated from target cohort, the phenotypic descriptions are relatively superficial and underrepresented and they may be inaccurate. Furthermore, very few genomic studies conducted fine-tuning steps to select the most accurate and optimized phenotyping approach for their study cohorts. As we know from plenty of previous studies, genetic heritability of complex human traits can be explained by a large number of genetic loci with small effects and there is significant genetic overlap among related human traits^5,6^. The highly accurate definition of human traits is critical for improving the power of obtaining causal inferences. To make the current genomic study more translational valuable, developing/selecting the optimized phenotyping method should be part of genomic study design from the very beginning.

However, how to define an accurate clinical phenotype for genetic modeling can be complicated due to the complexities of human traits and limitations of data mining methodologies^4,7–9^. Many genome-wide association studies (GWAS) use self-reported binary phenotypic descriptions or administrative data to establish phenotypes^10,11^. Prior research has shown that self-reported disease status and administrative data, such as billing data, are often inaccurate^12,13^. Applying a high-fidelity phenotyping method as part of GWAS pipeline is drawing lots of attention in the field.

The increasing use of EHR-based phenotypes in genomic research represented a new stage towards a more precise genome-phenome association study^8^. Compared with traditional self-reported phenotypes, EHR data can efficiently create standardized phenotypes with refinable definitions in large cohort studies. However, medical record data has a number of limitations. It has been designed primarily for clinical practice and has complicated formats, so to capture the various types of medical actions during patients’ diagnoses and treatments. For data mining, depending on the extraction methods, EHR information could be incomplete, inconsistent, or even conflict with each other, and can lead to different patients’ descriptions^9,14^. For example, the diagnosis for a diabetic patient can be drawn from their billing record, lab results such as HbA1c levels, or the clinical problem list, and these approaches will often yield conflicting results. Also, even though all type 1 diabetic patients will have an insulin treatment record, many type 2 diabetic patients will also have similar treatment data; this makes it hard to differentiate them without additional information. To extract high-quality phenotype information even in this instance which is relatively uncomplicated compared to many other distinctions (like that between COPD and asthma) requires careful consideration.

Several efforts have been made to further strengthen EHR phenotype accuracies by advanced informatics skills^15^. The Electronic Medical Records and Genomics (eMERGE) database is using both structured and unstructured data to create phenotypes through a series of filtering steps. Natural language processing (NLP) is applied to clinical text for addressing medical concept definition during this process. The Partners team also developed a phenotype algorithm recently which incorporate ICD code and NLP techniques to create a highly accurate phenotype^16–18^.

There have been multiple studies comparing different phenotyping approaches^19,20^. We previously showed that clinical problem lists from electronic health records are more specific than billing data, but not always sensitive for disease identification^14^. However, very few studies used genetic information to quantify the importance of accurate phenotyping. In this study, we used whole genome mutation patterns to evaluate three major clinical phenotyping methods:

1. Billing data extracted from a hospital finance system. These data are entered by clinicians or professional coders to substantiate charges to patients and their insurance companies, but is not routinely employed in clinical use. Past studies have shown that billing data are sensitive but not specific for disease identification^14,21^.
2. Clinical problem lists entered by healthcare providers in the EHR. These problem lists are longitudinal and frequently used during clinical care. Past studies have shown that problem lists are specific but not always sensitive^14,22^.
3. Curated phenotypes drawn from diverse EHR data, including problem lists, billing data, medications, laboratory results, clinical narratives (using natural language processing) and other EHR data. The so-called “phenotyping algorithm” was developed to use combinations of these EHR features to generate patient cohorts. These algorithms are designed to be more accurate than billing data or problem lists alone^14,16,23–25^.

## Results

### Polygenic risk score for different phenotypes

We selected 16,858 Caucasian subjects in 20 years medical record from the Partners Biobank who were genotyped using Illumina Multi-Ethnic Genotyping Array including 1,779,763 SNPs before quality control. We then applied a series of steps including linkage disequilibrium (LD) pruning to remove the low-quality SNPs before obtained 472,811 autosomal SNPs.

**Figure 1:**
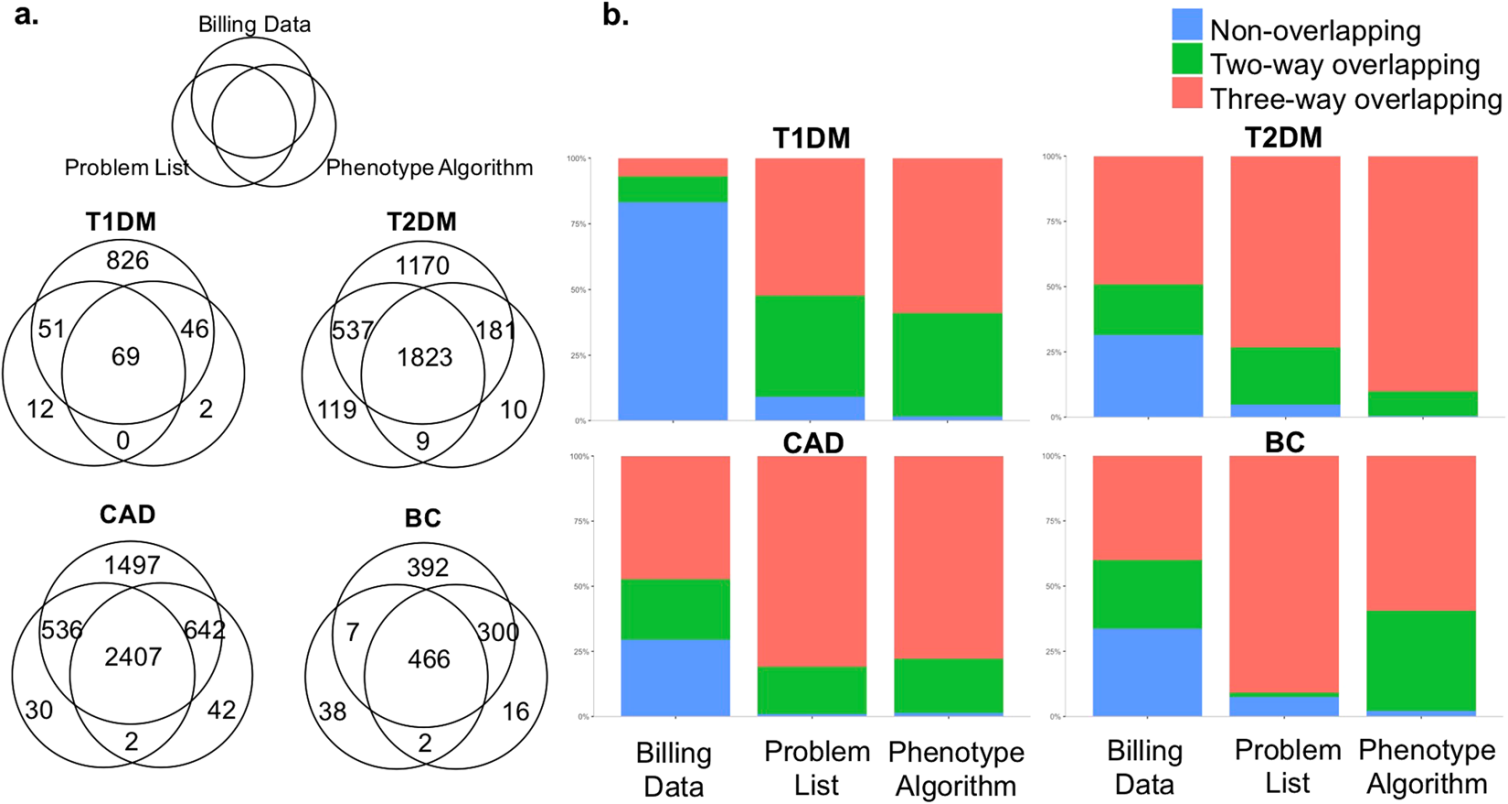
(**a**) The schematic diagram of patients identified by three different EHR extraction methods: Billing data alone, Problem List or Phenotype Algorithm. (**b**) The bar chart of percentages of patients in each phenotyping method: identified by only one specific phenotyping (Non-overlapping), by two phenotyping methods (Two-way overlapping), or by all three phenotyping methods (Three-way overlapping). For comparisons, there were 69, 1823, 2407 and 466 patients who recognized by all three methods in T1DM, T2DM, CAD and BC, which account for 7%, 49%, 47% and 40% of total billing code case populations. For phenotype algorithm, these percentages were 59%, 90%, 79% and 60%, while problem list approach had slightly lower percentages than phenotype algorithms. Meanwhile, there were 826, 1170, 1497 and 392 patients only discovered by billing code, converted to percentages of 83%, 32%, 29% and 34%, while both problem list and phenotype algorithm had only few patients uniquely identified. T1DM: type1 diabetes mellitus; T2DM: type2 diabetes mellitus; CAD: coronary artery disease; BC: breast cancer.

We then extracted phenotyping data from the Partners Biobank, the Partners Research Patient Data Registry (RPDR) and Enterprise Data Warehouse (EDW). Four complex diseases with different genetic heritabilities were chosen for this study: type 1 diabetes mellitus (T1DM), type 2 diabetes mellitus (T2DM), coronary artery disease (CAD) and breast cancer (BC). For each disease, we obtained the full clinical records so different phenotype extractions can be applied to the whole study cohort for comparison. We then used three phenotyping methods to identify the case cohort: the billing data alone, problem list and a Partners team developed phenotype algorithm (Table 1, Extended Data Table 1)^16^. In all diseases, using billing data along, we identified most patients and a significant portion of them were not recognized by the other two methods (Fig. 1).

**Table 1:** Summary of three phenotype extraction methods.

Phenotype Methods	Description	Data Source Structure	Original Purpose	Whether or not reviewed by Physicians	Merits/Shortcomings
Billing Data	the code system created for recording all the actions need insurance payments	structured data	insurance reimbursements	No	high sensitivity, low specificity
Problem List	temporal record of important problems happed to patients	structured data	diagnosis	Yes	low sensitivity, high specificity
Phenotype Algorithm	calculated phenotype based on the combination of ICD code and NLP processed patient notes	structured data + un-structured data	phenotype extraction	No	balanced method between sensitivity and specificity

For each disease, we used three EHR-derived phenotyping methods to identify the case cohort: the billing data, clinical problem list and a Partners team developed phenotype algorithm.

In order to compare the three phenotyping methods, we developed a genetic model to predict whether patients had the disease of interest. We chose a polygenic approach, because multiple studies have showed the limitation of individual-SNP association models which can only explain small amount phenotypic variations and then cause so-called “missing heritability”^26^. Recently, the polygenic risk score (PRS), a statistic summarizing association weights from multiple genetic loci, was proved to have strong predictive power for common complex diseases^27^. The four diseases in our study were all shown to be associated with multiple genetic mutations in prior studies^5,28^. We therefore used polygenic scores association models based on three phenotyping methods respectively, to conduct comparison.

Using 472,811 SNPs as dependent variables and case/control groups from each phenotype (Extended Data Table 1), a logistic regression was trained and odds ratio for each SNP was obtained. We used the components from principal component analysis (PCA) to adjust for population structure within the Caucasian ancestry. Both q-q plots and genomic inflation factor showed no systematic inflation (Extended Data Table 2, Extended Data Fig. 1). The 5-fold cross-validation was performed to develop and evaluate polygenic scores. We calculated PRS for each individual in testing sets based on these SNPs’ odds ratio and allele frequency. Using a series of p values as cutoffs, the SNP selection was then performed to identify the best SNP subsets for PRS calculation for each phenotype.

### The phenotype algorithm leads to a more accurate genomic prediction model

With the generated PRS, we first compared the score mean differences between control and case groups to estimate its discriminatory power (Table 2). For T1DM, the billing data alone phenotype generated a mean difference of 0.00054, reflecting only a slight difference between the groups. However, we obtained 0.04516 for phenotype algorithm approach, indicating a more separated control and case distributions. For problem list, the difference was 0.00211, still better than billing data alone.

**Table 2:** Summary of genetic evaluations for three phenotypes.

Disease	Phenotype	PRS Mean Difference (Case-Control) (S.D.)	AUC (S.D.)	Odds Ratio (S.D.)
T1DM	Billing Data	0.00054 (0.00003)	0.5480 (0.0170)	1.18 (0.06)
	Problem List	0.00211 (0.00053)	0.6378 (0.0314)	1.33 (0.09)
	Phenotype Algorithm	0.04516 (0.00783)	0.7012 (0.0318)	1.85 (0.07)
T2DM	Billing Data	0.00010 (0.00002)	0.5473 (0.0063)	1.24 (0.05)
	Problem List	0.00028 (0.00004)	0.5854 (0.0067)	1.32 (0.06)
	Phenotype Algorithm	0.00062 (0.00005)	0.5878 (0.0038)	1.41 (0.04)
CAD	Billing Data	0.00299 (0.00025)	0.5604 (0.0049)	1.22 (0.06)
	Problem List	0.00437 (0.00019)	0.5973 (0.0095)	1.35 (0.06)
	Phenotype Algorithm	0.01206 (0.00031)	0.6249 (0.0041)	1.68 (0.03)
BC	Billing Data	0.00006 (0.000006)	0.5291 (0.0029)	1.07 (0.02)
	Problem List	0.00009 (0.000003)	0.5382 (0.0052)	1.11 (0.07)
	Phenotype Algorithm	0.00015 (0.00002)	0.5681 (0.0039)	1.20 (0.03)

The summary table for PRS score mean differences between control and case groups in four diseases with three EHR phenotype extraction methods. Also, the odds ratios from logistic regression were obtained. The predictive performances of polygenic models were estimated by the area under the curve (AUC) values. PRS: polygenic risk score Odds Ratio: odds ratio per standard deviation increase.

In T2DM, CAD and BC, we observed the similar pattern with the phenotype algorithm had the larger mean differences than billing data. In all the cases, the problem list method had a better separation than billing data, but worse than phenotype algorithms.

The mean differences can only suggest the differential power of these scores, but we wanted to further compare the performances of these phenotyping methods as patient classifiers. The receiver operating characteristic curve (ROC curve) is a widely-used tool to measure the diagnostic ability of a binary classifier. It plots different cutoffs to discover the best balance point between discriminatory sensitivity and specificity. Based on that, the area under the curve (AUC) value is the single numeric summary to measure the classifier’s performance. We then plotted ROC curve and calculated AUCs for all sets of PRSs to evaluate them.

**Figure 2:**
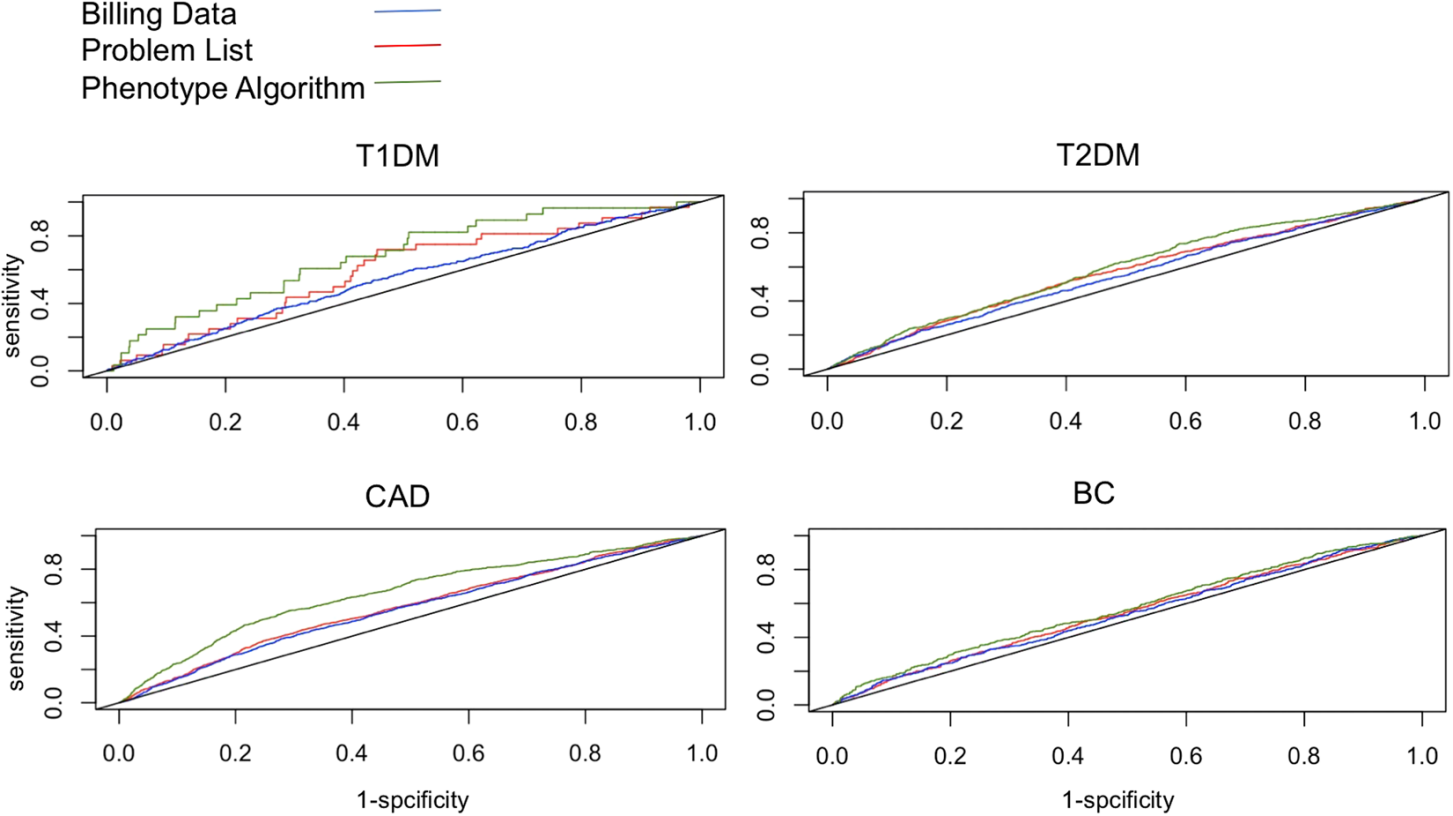
The receiver operating characteristic curve (ROC curve) for polygenic models in four diseases using three different EHR phenotype extraction methods.

By ROC and AUC, we observed a clear pattern among these models (Table 2 and Fig. 2). For each disease, the phenotyping algorithm led to the most accurate polygenic model, with AUCs of 0.70, 0.59, 0.62 and 0.57 for T1DM, T2DM, CAD and BC, respectively. The billing data alone performed worse for each disease, with AUCs of 0.55, 0.55, 0.56 and 0.53. We also calculated odds ratio per standard deviation increase for these models^29^, and the same ranking was reflected. The problem list had a slightly lower discriminatory ability than phenotype algorithm in all the cases, but still better than billing data alone.

Most of the current genome-wide association studies have the discriminatory ability of low to modest for most of the common complex diseases, with AUC values of less than 60% or 70%, which fits with PRS scales from our models^30,31^. The better performance of T1DM than the other three diseases, due to its high heritability and relatively low prevalence, was also consistent with previous studies^32^.

In order to prove the generality of our results, we also tested statistical summaries from recently published GWAS studies for each disease^33–36^. Using the odds ratio from these independent datasets, we generated PRS in our cohort for each phenotype. Using ROC curve and AUC values, we evaluated three phenotypes again. The similar ranking among them was seen (Table 3). In other words, even using models trained on other data, model performance is greater when more accurate phenotypes are used.

**Table 3:** Summary of genetic evaluations for three phenotypes using published odds ratio.

Disease	Phenotype	PRS Mean Difference (Case-Control)	AUC	Odds Ratio
T1DM	Billing Data	0.00056	0.5381	1.12
	Problem List	0.00329	0.6219	1.39
	Phenotype Algorithm	0.00619	0.7212	1.83
T2DM	Billing Data	0.00012	0.5439	1.19
	Problem List	0.00018	0.5632	1.38
	Phenotype Algorithm	0.00021	0.5821	1.38
CAD	Billing Data	0.00012	0.5639	1.42
	Problem List	0.00029	0.5891	1.44
	Phenotype Algorithm	0.00037	0.6372	1.61
BC	Billing Data	0.00052	0.5339	1.42
	Problem List	0.00058	0.5613	1.47
	Phenotype Algorithm	0.00061	0.5701	1.61

The summary table for PRS score mean differences between control and case groups in four diseases. We used the statistics summaries from published GWAS studies for these diseases to calculate PRS. Also, the odds ratios from logistic regression were obtained. The predictive performances of polygenic models were estimated by the area under the curve (AUC) values. PRS: polygenic risk score, Odds Ratio: odds ratio per standard deviation increase.

### Billing data phenotype can be improved

Unlike problem lists, which are longitudinal, billing data can repeat each time a patient encounters the healthcare system and is charged for additional care. Past studies have shown that patients who have been billed multiple times for a particular disease are more likely to have that disease than those billed only once^37^. We studied the effect of setting a higher threshold for classifying a patient as disease-affected by identifying subsets of patients billed at least once, at least twice or at least three times for each of the four diseases we were interested in. This approach may increase specificity at a possible cost to sensitivity.

In all four diseases, compared with patients billed only once for the disease, the subsets of patients with at least 2 or 3 billing occurrences had much higher percentages for the regions shared by all three phenotypes. For example, in T2DM, the percentages of patients identified by all the methods accounted for 49%, 64% and 72% of billing data alone patients with at least 1, 2 or 3 visits respectively (Extended Data Fig. 2).

Using the same p value cutoffs used for whole billing code patients, we generated PRS for these subsets before plotting ROC curve. As expected, compared with the whole billing code population, the AUC values improve in subsets of patients with multiple bills for each disease (Table 4). However, the AUC was still lower than the curated phenotype or problem list.

**Table 4:** Summary of genetic evaluations for billing code sub-phenotypes.

Disease	Phenotype	PRS Mean Difference (Case-Control) (S.D.)	AUC (S.D.)	Odds Ratio (S.D.)
T1DM	Billing_visit1	0.00054 (0.00003)	0.5480 (0.0170)	1.18 (0.06)
	Billing_visit2	0.00138 (0.00007)	0.5689 (0.0210)	1.26 (0.05)
	Billing_visit3	0.00153 (0.00008)	0.5512 (0.0190)	1.26 (0.09)
T2DM	Billing_visit1	0.00010 (0.00002)	0.5473 (0.0063)	1.24 (0.05)
	Billing_visit2	0.00014 (0.00003)	0.5666 (0.0048)	1.25 (0.03)
	Billing_visit3	0.00013 (0.00001)	0.5656 (0.0039)	1.25 (0.05)
CAD	Billing_visit1	0.00299 (0.00025)	0.5604 (0.0049)	1.22 (0.06)
	Billing_visit2	0.00475 (0.00031)	0.5749 (0.0042)	1.31 (0.05)
	Billing_visit3	0.00472 (0.00019)	0.5779 (0.0039)	1.31 (0.09)
BC	Billing_visit1	0.00006 (0.000006)	0.5291 (0.0029)	1.07 (0.02)
	Billing_visit2	0.00009 (0.000003)	0.5390 (0.0017)	1.12 (0.03)
	Billing_visit3	0.00009 (0.000008)	0.5293 (0.0023)	1.10 (0.05)

The summary table for PRS score mean differences between control and case groups in four diseases. We subset the billing data patient cohorts according to their hospital visiting times: subset for patients with at least 1 time visit, 2 times visits or 3 times visits. Also, the odds ratios from logistic regression were obtained. The predictive performances of polygenic models were estimated by the area under the curve (AUC) values. PRS: polygenic risk score, Billing_visit1: Billing code patients with at least one visit, Billing_visit2: Billing code patients with at least two visits, Billing_visit3: Billing code patients with at least three visits.

## Discussion

With the development of high throughput genomic technology, researchers are now able to explore the genetic variations in large-scale populations. In genetically related disorders such as diabetes, heart diseases and cancer, genome-wide association studies have identified numerous genetic mutation sites which are highly associated with these diseases^36,38,39^. This is becoming the foundation for precision medicine and gene therapies, which have the potential to revolutionize medical therapy. In last 10 years, high quality genomic data are becoming widely available, including the 1000 Genome Project, the ENCODE Project, and Roadmap Epigenomics Project, among others. With these huge efforts, we now have a much more comprehensive and complete picture of human genome structure and related functional modules. As of 2015, more than 15,000 SNPs had been found to be associated with complex human traits, suggesting a promising future for broad clinical application^40^. With the deeper understanding of the relationship between genomic architecture and complex human traits, genetic variants need to be linked with accurate phenotype descriptions to develop causal genome-phenome associations.

Although phenotyping algorithms are now available, many GWAS use only simple phenotyping data^41^. For example, the UK Biobank, a data source for many important genomic studies, is using questionnaire and administrative data as its main diagnosis standard. Also, the billing data is the main phenotyping tool in phenome-wide association studies (PheWASs). Although the billing data alone has been shown to be efficient to identify patients, some recent studies, however, illustrated it can be less accurate^42–44^.

Further, in order to increase the statistical power of GWAS, meta-analysis has been widely used, in which multiple study cohorts from different databases are combined to achieve a larger sample size. These cohorts could come from different countries, using different platforms, or may have been identified by different institutes, which all can lead to the heterogeneities of phenotype definitions^45,46^.

The rich information in current EHR system makes it possible to generate accurate, repeatable and costumed disease definition. Multiple EHR phenotyping tools, such as PheNorm and the phenotype algorithms developed by eMERGE network and Partners Biobank, were showed to be able to facilitate standardized phenotyping in large-scale cohort^16,17,23^. By selecting the optimized EHR-based phenotype, the more targeted genomic study can be achieved. Due to complicated EHR phenotype assembly process and various phenotype data densities in different genomic studies, the evaluation tools which can measure and estimate the performance of different phenotype extraction methods would be useful for future genomic studies.

We tried to link a polygenic association model with different EHR phenotype extractions. The polygenic risk score was shown to be able to explain high percentages of genetic variations for complex traits and was also used to estimate the genetic correlations among traits. Although the AUCs achieved by this model is still not matching the clinical expectation, we were able to apply it for clinical phenotyping comparisons. In multiple complex diseases, using both our data and published statistic summaries, the polygenic score quantitatively reflected the performances of different phenotype extraction methods, which is also consistent with previous studies. With the increasing needs to integrate patient genetic profile and clinical data to improve the current precision medicine, we provided some useful explorations in how to cross-validate these two sides of patient information.

With the comparative studies performed here, we tried to demonstrate several key points to facilitate the future integrated researches between genomic information and patient record data. Firstly, a careful review is needed for EHR phenotypes. As we and others have showed, each EHR extraction has its advantages and shortcomings. A sensitivity/specificity balanced approach is critical for high-fidelity phenotypes. The billing data alone, with their high sensitivity, identified the most patients, while the problem list or phenotype algorithm only discovered very few unique patients. However, the performance of billing data model is the worst among three, suggesting a high false-positive rate for those “billing data-only” patients. Secondly, for EHR phenotype extraction, a strong definition of disease is critical. The problem list phenotype, with its key function to identify the most relevant issues with patients, showed the better discriminatory ability than billing data alone phenotype. Also, in our improved version of billing data phenotype, we subset the patients with multiple visits, so to “strengthen” the probability of real disease for those patients. As showed in our results, the subset patients were more “concentrated” in the common regions shared with the other two phenotypes. This could be a useful trick for GWAS researchers, as the billing data is currently one of the most used EHR phenotype in the field. By using the more “defined” billing data identified patients, a more accurate phenotype could be achieved. Similarly, other strategies could be used to obtain a more confirmed disease definition, including using multiple ICD codes per patient to confirm diseases, combined with other codified data such as CPT-4 and LOINC, and combined with NLP results. Lastly, a phenotype “tuning” process could be really useful for large scale association studies. To our knowledge, most of current GWAS studies have limited descriptions about how phenotypes are generated and what related parameters are, therefore make these huge research efforts somewhat blindly. There is also lack of useful tool to easily quantify the potential phenotype “contaminations”. As we showed here, phenotype extraction requires the deep understanding of clinical data structures and is a pipeline with multiple steps. To generate high degree of accuracy for phenotype inference needs the optimization of each step and careful integration of different data elements.

This study has several limitations. First, the sample sizes of T1DM patient for problem list and phenotype algorithm were relatively small, which could cause the imbalance of case and control and might not be sufficient to represent the whole distribution. The relative small values of genomic inflation factor for these two phenotypes also suggested the potential deflation. Second, due to the limitation of research expenses, we did not apply chart review to establish the “gold standard” for disease identification. But through the independent published datasets, we confirmed the performances ranking of three phenotyping methods. We are therefore confident about the patterns we observed in our dataset. As another limitation, we only used common SNPs for PRS calculations. It was previously shown that rare genetic variations could also contribute to the phenotypes of these diseases^47^. For the future work, we will enlarge the sample size to gain more statistic power of the analysis. Deeper clinical data mining, including chart review, will also be conducted to optimize the phenotyping process. For PRS calculation, imputation will be performed on genotype data to identify rare variants.

## Methods

### Clinical databases

Three large clinical databases were utilized for this study, including Partners Biobank, Partners Research Patient Data Registry (RPDR) and Enterprise Data Warehouse (EDW).

Partners Biobank is a large integrated database for different types of biomedical data^18^. It includes medical information of ~75,000 patients from multiple Harvard affiliated hospitals. All these patients have given their consent for broad-based research. Facilitated with powerful searching engine, the high quality clinical data and survey data on lifestyle, environment and family history are available in the database. Also, there is matched genomic data for ~20,000 subjects in the database. RPDR is a centralized clinical data registry/warehouse. It collects data from hospital EHR system and stores it in single database for research purposes. EDW is a central clinical database which contains all the patients’ information from hospital medical data system. Various of patients’ information can be matched across these three databases through enterprise master patient index (EMPI) numbers. In this study, we extracted genetic and clinical phenotypic information for 16,858 Caucasian subjects from these databases and match them for genome-phenome association study.

All participants signed a consent prior to study participation. The study’s protocol was reviewed and approved by Partners Human Research Committee under the IRB number 2017P000327/PHS.

### Diseases phenotype data

The clinical phenotype data of study cohort from 1987 to 2017 was obtained from Partners Biobank, RPDR and EDW data warehouses, including patients from Brigham and Women’s Hospital (BWH), Massachusetts General Hospital, McLean Hospital (MCL) and four other hospitals in Partners System, which are all sharing the centralized clinical database. We acquired all the medical diagnosis information for the study cohort, which contains 11,820,720 rows of records, including patient’s EMPI number, the billing data diagnosis, problem list diagnosis, patient visiting date record and diagnosis results from phenotype algorithm. We then created the phenotype data table for four diseases and extracted three different phenotypes for each disease.

#### Billing data phenotype

For billing data phenotype, we used both ICD9 and ICD10 codes corresponding with T1DM, T2DM, CAD and BC respectively, to query phenotypes data table. All the codes used were manually reviewed to filter out un-specific descriptions, such as disease due to underlying condition, drug or chemical induced disease and postprocedural diseases. We include diseases of interest with or without different complications. The pre-existing conditions were also included in our cohorts. Below is the list of codes used for each disease:

- T1DM
  ICD9: 250.01, 250.03, 250.11, 250.13, 250.21, 250.23, 250.31, 250.33, 250.41, 250.43, 250.51, 250.53, 250.61, 250.63, 250.71, 250.73, 250.81, 250.83, 250.91, 250.93
  ICD10: E10.*, O24.0
- T2DM
  ICD9: 250.00, 250.02, 250.10, 250.12, 250.20, 250.22, 250.30, 250.32, 250.40, 250.42, 250.50, 250.52, 250.60, 250.62, 250.70, 250.72, 250.80, 250.82, 250.90, 250.92
  ICD10: E11.*
- CAD
  ICD9: 414.0, 414.2, 414.3, 414.4, 414.8, 414.9, 412
  ICD10: I25.*, I21.*
- BC

   ICD9: 174.*

   ICD10: C50.*

#### Problem list phenotype

For problem list phenotype, we used physician reviewed problem list records from both centralized Epic system (Epic Systems Corporation) and Partners developed systems, including Longitudinal Medical Record (LMR) and OnCall Record. We created the key word lists of clinical symptoms directly related with these diseases. The phenotype data table was queried with these lists to identify the case and control cohorts, the key descriptions for each disease were described below:

- T1DM: Diabetes mellitus type 1, Diabetes mellitus (juvenile onset), Diabetes mellitus insulin dependent and Type 1 diabetes with different complications.
- T2DM: Diabetes mellitus type 2, Diabetes mellitus (adult onset), and Type 2 diabetes with different complications.
- CAD: Coronary artery disease, Myocardial infarction, Acute myocardial infarction and CAD with different procedures.
- BC: Breast cancer, Breast cancer ductal *in situ*, Breast cancer lobular *in situ* and Breast carcinoma.

#### Phenotype algorithm

A phenotype algorithm was developed by Partners Portal team previously using structured data and unstructured data. Nature Language Processing (NLP) was applied to extract information from narrative text to increase the prediction power. For each disease, the chart review was conducted by physicians to establish the gold standard. During the process, the most relevant features were identified by automated feature extraction protocol (AFEP), including comorbidities, symptoms and medications, etc. Based on the frequencies of these features in patients notes, a further screen was performed. ICD, CPT-4 and LOINC code were used to represent the coded features. A logistic regression classifier was then developed using these features. To further optimize the model, the adaptive least absolute shrinkage and selection operator (LASSO) procedure was used to identify most important features for the final model. Below are the features used for different diseases in our study:

- T1DM: billing code diagnosis for T1DM, dyslipidemia and hypoglycemia; count of prescriptions for cortical steroid, insulin, insulin aspart, insulin lispro, and metformin; BMI, and total number of visits with a coded diagnosis.
- T2DM: billing code diagnosis for T2DM, NLP extracted features including creatinine, diabetes, hormones, and metformin.
- CAD: billing code diagnosis for CAD and ischemic heart disease; NLP extracted features including alcohol, angioplasty, antiplatelet agents, coronary artery bypass grafting, coronary atherosclerosis, coronary heart disease, creatinine, electrocardiogram, ischemia, ischemic cardiomyopathy, myocardial infarction, nitroglycerin, and platelet aggregation inhibitors
- BC: count of coded diagnosis of breast cancer

We obtained the cohorts for phenotype algorithm through curated diagnosis sections in Partners Biobank. The patients with positive predictive value of 0.90 for these diseases were used to generate the case cohorts^16^.

All EMPI number from EHR record were converted to Biobank ID to link with patients’ genomic data.

### Genotyping data and genome-wide association analysis

The genotyping was performed by Partners Biobank using Illumina’s Multi-Ethnic array including 1,779,763 SNPs. The under-performing SNPs were removed during quality control (QC) process conducted by Biobank’s team using Illumina’s manual clustering process. The annotation was conducted using Alamut Batch, a commercial software developed by Interactive Biosoftware. Using these data files, we performed additional QC steps to further improve data quality. The detail procedure was described previously^48^.

First, we only selected Caucasian population to avoid bias induced by population stratification. Using principal components analysis plot, we also confirmed that there was no significant population stratification^49^.

Second, we selected autosomal SNPs with minor allele frequency (MAF) greater than 1% and genotyping rate 90% or greater to further remove bad SNPs. We also checked the Hardy-Weinberg Equilibrium (HWE) and removed failed SNPs. Three rounds of linkage disequilibrium (LD) pruning processes were then conducted to remove SNPs in approximate linkage equilibrium. The parameters of window size, variant count to shift the window and variance inflation factor were 50, 5 and 10. After this step, there were 472,811 SNPs remaining.

Third, we conducted principal component analysis (PCA) among SNPs and used the first two components to control for population stratification within Caucasian population.

For each disease, we then generated multiple fam files using case/control groups identified by three phenotyping methods. The logistic regressions were then performed for each fam file. Q-Q plots and genome inflation factors (λgc) were obtained to monitor systematic biases in association results^50^.

### Polygenic score

The detail of the polygenic score model was described elsewhere^1,27^. Briefly, we obtained the odds ratio for all SNPs through logistic regression for each phenotype, then subsets of SNPs were selected by using different p values as cutoffs. We tested p values from 0.00005 to 0.1 in each phenotype and obtained 12 subsets sized from ~30 to ~50,000 SNPs, respectively. The individual PRS in testing set was calculated using each SNP subset and the AUC of each subset was calculated and compared. We then used optimized SNP subset to calculate risk score for each individual in testing set by summing up across SNPs the risk allele frequencies multiplied by the logarithm of the odds ratio. The ROC curve was then plotted for different score cutoffs to evaluate the performance of the models. The same SNP subsets selection process was applied to the published GWAS datasets for four diseases.

### Statistical analysis and software

The genome-wide logistic regression was performed by using PLINK 1.90^51^. All other analyses were conducted by R (version 3.3.3). We used 5-fold cross-validation to prevent overfitting in our dataset: for each fold, the full dataset was split into 80% and 20%: 80% as training set to calculate odds ratio for each SNP and 20% as the testing set to calculate the polygenic score for each individual. This procedure was repeated for 5 times. For different phenotypings in each disease, the same training and testing subgroups were used so the performances can be compared. The polygenic score was calculated by using the –score functions in PLINK. The log odds ratio produced from logistic regression was used as the association weight.

### Data availability

The datasets generated and/or analyzed during the current study are not publicly available due to IRB regulation. The summary statistics are available from the corresponding author on reasonable request.

## Electronic supplementary material

Supplementary Material

## References

[1] Yang J (2010). Common SNPs explain a large proportion of the heritability for human height. Nat Genet.

[2] Horikoshi M (2016). Genome-wide associations for birth weight and correlations with adult disease. Nature.

[3] Huang H (2017). Fine-mapping inflammatory bowel disease loci to single-variant resolution. Nature.

[4] Murphy S (2009). Instrumenting the health care enterprise for discovery research in the genomic era. Genome Res.

[5] Wellcome Trust Case Control, C. Genome-wide association study of 14,000 cases of seven common diseases and 3,000 shared controls. *Nature* **447**, 661–678, 10.1038/nature05911 (2007). 10.1038/nature05911 PMC2719288 17554300

[6] Bulik-Sullivan B (2015). An atlas of genetic correlations across human diseases and traits. Nat Genet.

[7] Kohane IS (2011). Using electronic health records to drive discovery in disease genomics. Nat Rev Genet.

[8] Hripcsak G, Albers DJ (2017). High-fidelity phenotyping: richness and freedom from bias. J Am Med Inform Assoc.

[9] Wei WQ, Denny JC (2015). Extracting research-quality phenotypes from electronic health records to support precision medicine. Genome Med.

[10] Mersha TB, Abebe T (2015). Self-reported race/ethnicity in the age of genomic research: its potential impact on understanding health disparities. Hum Genomics.

[11] Grams ME (2014). Performance and limitations of administrative data in the identification of AKI. Clin J Am Soc Nephrol.

[12] Oksanen T (2010). Self-report as an indicator of incident disease. Ann Epidemiol.

[13] Powell H, Lim LL, Heller RF (2001). Accuracy of administrative data to assess comorbidity in patients with heart disease. an Australian perspective. J Clin Epidemiol.

[14] Wright A (2011). A method and knowledge base for automated inference of patient problems from structured data in an electronic medical record. J Am Med Inform Assoc.

[15] Krishnamoorthy P, Gupta D, Chatterjee S, Huston J, Ryan JJ (2014). A review of the role of electronic health record in genomic research. J Cardiovasc Transl Res.

[16] Liao KP (2015). Development of phenotype algorithms using electronic medical records and incorporating natural language processing. BMJ.

[17] Gottesman O (2013). The Electronic Medical Records and Genomics (eMERGE) Network: past, present, and future. . Genet Med.

[18] Gainer, V. S. *et al*. The Biobank Portal for Partners Personalized Medicine: A Query Tool for Working with Consented Biobank Samples, Genotypes, and Phenotypes Using i2b2. *J Pers Med* **6**, 10.3390/jpm6010011 (2016). 10.3390/jpm6010011 PMC4810390 26927184

[19] Wei WQ (2016). Combining billing codes, clinical notes, and medications from electronic health records provides superior phenotyping performance. J Am Med Inform Assoc.

[20] Chen CY (2018). Genetic validation of bipolar disorder identified by automated phenotyping using electronic health records. Transl Psychiatry.

[21] Wright A, Chen ES, Maloney FL (2010). An automated technique for identifying associations between medications, laboratory results and problems. J Biomed Inform.

[22] Wright A, Maloney FL, Feblowitz JC (2011). Clinician attitudes toward and use of electronic problem lists: a thematic analysis. BMC Med Inform Decis Mak.

[23] Yu S (2017). Enabling phenotypic big data with PheNorm. J Am Med Inform Assoc.

[24] Ritchie MD (2010). Robust replication of genotype-phenotype associations across multiple diseases in an electronic medical record. Am J Hum Genet.

[25] Newton KM (2013). Validation of electronic medical record-based phenotyping algorithms: results and lessons learned from the eMERGE network. J Am Med Inform Assoc.

[26] Manolio TA (2009). Finding the missing heritability of complex diseases. Nature.

[27] International Schizophrenia C (2009). Common polygenic variation contributes to risk of schizophrenia and bipolar disorder. Nature.

[28] Machiela MJ (2011). Evaluation of polygenic risk scores for predicting breast and prostate cancer risk. Genet Epidemiol.

[29] Lall K, Magi R, Morris A, Metspalu A, Fischer K (2017). Personalized risk prediction for type 2 diabetes: the potential of genetic risk scores. Genet Med.

[30] Potenciano V, Abad-Grau MM, Alcina A, Matesanz F (2016). A comparison of genomic profiles of complex diseases under different models. BMC Med Genomics.

[31] Chatterjee N, Shi J, Garcia-Closas M (2016). Developing and evaluating polygenic risk prediction models for stratified disease prevention. Nat Rev Genet.

[32] Wray NR, Yang J, Goddard ME, Visscher PM (2010). The genetic interpretation of area under the ROC curve in genomic profiling. PLoS Genet.

[33] Speed D (2017). Reevaluation of SNP heritability in complex human traits. Nat Genet.

[34] Webb TR (2017). Systematic Evaluation of Pleiotropy Identifies 6 Further Loci Associated With Coronary Artery Disease. J Am Coll Cardiol.

[35] Replication DIG (2014). Genome-wide trans-ancestry meta-analysis provides insight into the genetic architecture of type 2 diabetes susceptibility. Nat Genet.

[36] Michailidou K (2017). Association analysis identifies 65 new breast cancer risk loci. Nature.

[37] Dasenbrock HH (2017). Validation of an International Classification of Disease, Ninth Revision coding algorithm to identify decompressive craniectomy for stroke. BMC Neurol.

[38] Fuchsberger C (2016). The genetic architecture of type 2 diabetes. Nature.

[39] International Consortium for Blood Pressure Genome-Wide Association, S. *et al*. Genetic variants in novel pathways influence blood pressure and cardiovascular disease risk. *Nature* **478**, 103–109, 10.1038/nature10405 (2011). 10.1038/nature10405 PMC3340926 21909115

[40] Lowe WL, Reddy TE (2015). Genomic approaches for understanding the genetics of complex disease. Genome Res.

[41] Korte A, Farlow A (2013). The advantages and limitations of trait analysis with GWAS: a review. Plant Methods.

[42] Tu K, Mitiku T, Guo H, Lee DS, Tu JV (2010). Myocardial infarction and the validation of physician billing and hospitalization data using electronic medical records. Chronic Dis Can.

[43] Tu K, Mitiku T, Lee DS, Guo H, Tu JV (2010). Validation of physician billing and hospitalization data to identify patients with ischemic heart disease using data from the Electronic Medical Record Administrative data Linked Database (EMRALD). Can J Cardiol.

[44] Kern EF (2006). Failure of ICD-9-CM codes to identify patients with comorbid chronic kidney disease in diabetes. Health Serv Res.

[45] Zeggini E, Ioannidis JP (2009). Meta-analysis in genome-wide association studies. Pharmacogenomics.

[46] Ioannidis JP, Patsopoulos NA, Evangelou E (2007). Heterogeneity in meta-analyses of genome-wide association investigations. PLoS One.

[47] Jun G (2018). Evaluating the contribution of rare variants to type 2 diabetes and related traits using pedigrees. Proc Natl Acad Sci USA.

[48] Turner, S. *et al*. Quality control procedures for genome-wide association studies. *Curr Protoc Hum Genet* **Chapter 1**, Unit1 19, 10.1002/0471142905.hg0119s68 (2011). 10.1002/0471142905.hg0119s68 PMC3066182 21234875

[49] Price AL (2006). Principal components analysis corrects for stratification in genome-wide association studies. Nat Genet.

[50] Devlin B, Roeder K (1999). Genomic control for association studies. Biometrics.

[51] Purcell S (2007). PLINK: a tool set for whole-genome association and population-based linkage analyses. Am J Hum Genet.

