# Supplementary material for: Using whole genome scores to compare three clinical phenotyping methods in complex diseases
Source: Sci Rep. 2018 Jul 27;8:11360. doi: 10.1038/s41598-018-29634-w (PMC6063939; doi:10.1038/s41598-018-29634-w)
Supplement: Supplementary file 1 — Supplementary Material [file 41598_2018_29634_MOESM1_ESM.docx]

**Using whole genome scores to compare three clinical phenotyping methods in complex diseases**

Wenyu Song, Ph.D.^1, 2^, Hailiang Huang, Ph.D.^3,5^, Cheng-Zhong Zhang, Ph.D.^2,4,5^, David W. Bates, M.D., MSc. ^1, 6^, Adam Wright, Ph.D.* ^1, 2, 6^

^1^Division of General Internal Medicine and Primary Care, Brigham and Women's Hospital and Harvard Medical School, Boston, Massachusetts, 02120, USA; ^2^Department of Biomedical Informatics, Harvard Medical School, Boston, Massachusetts, 02115, USA;

^3^Analytic and Translational Genetics Unit, Massachusetts General Hospital and Harvard Medical School, Boston, Massachusetts, 02114, USA; ^4^Department of Biostatistics and Computational Biology, Dana-Farber Cancer Institute and Harvard Medical School, Boston, Massachusetts, 02215, USA; ^5^Broad Institute of MIT and Harvard, Cambridge, Massachusetts, 02142, USA; ^6^ Information Systems Department, Partners HealthCare, Somerville, Massachusetts, 02145, USA. *Correspondence to [AWRIGHT@BWH.HARVARD.EDU]

**Extended data figures and tables**

**Extended Data Table1. Characteristic summary table for each disease**

| Disease | Phenotype | Cases | Controls | Total Number |
| --- | --- | --- | --- | --- |
| T1DM | Billing Data | 992 | 15866 | 16858 |
|  | Problem List | 132 | 16726 | 16858 |
|  | Phenotype Algorithm | 117 | 16741 | 16858 |
| T2DM | Billing Data | 3711 | 13147 | 16858 |
|  | Problem List | 2488 | 14370 | 16858 |
|  | Phenotype Algorithm | 2023 | 14835 | 16858 |
| CAD | Billing Data | 5082 | 11776 | 16858 |
|  | Problem List | 2975 | 13883 | 16858 |
|  | Phenotype Algorithm | 3093 | 13765 | 16858 |
| BC | Billing Data | 1165 | 15693 | 16858 |
|  | Problem List | 513 | 16345 | 16858 |
|  | Phenotype Algorithm | 784 | 16074 | 16858 |

**Extended Data Table1.**This is a summary table of number of case and control subjects in all four diseases with different phenotyping methods.

**Extended Data Table2. Genomic Inflation Factor (λgc)**

|  | T1DM | T2DM | CAD | BC |
| --- | --- | --- | --- | --- |
| Billing Data | 1.011 | 1.033 | 1.027 | 1.008 |
| Problem List | 1.020 | 1.031 | 1.034 | 1.008 |
| Phenotype Algorithm | 0.991 | 1.034 | 1.041 | 1.010 |

**Extended Data Table2.** Genomic Inflation factor for logistic regression models in four diseases using three different EHR phenotype extraction methods.

**Extended Data Figure1. Q-Q plots summary**

**
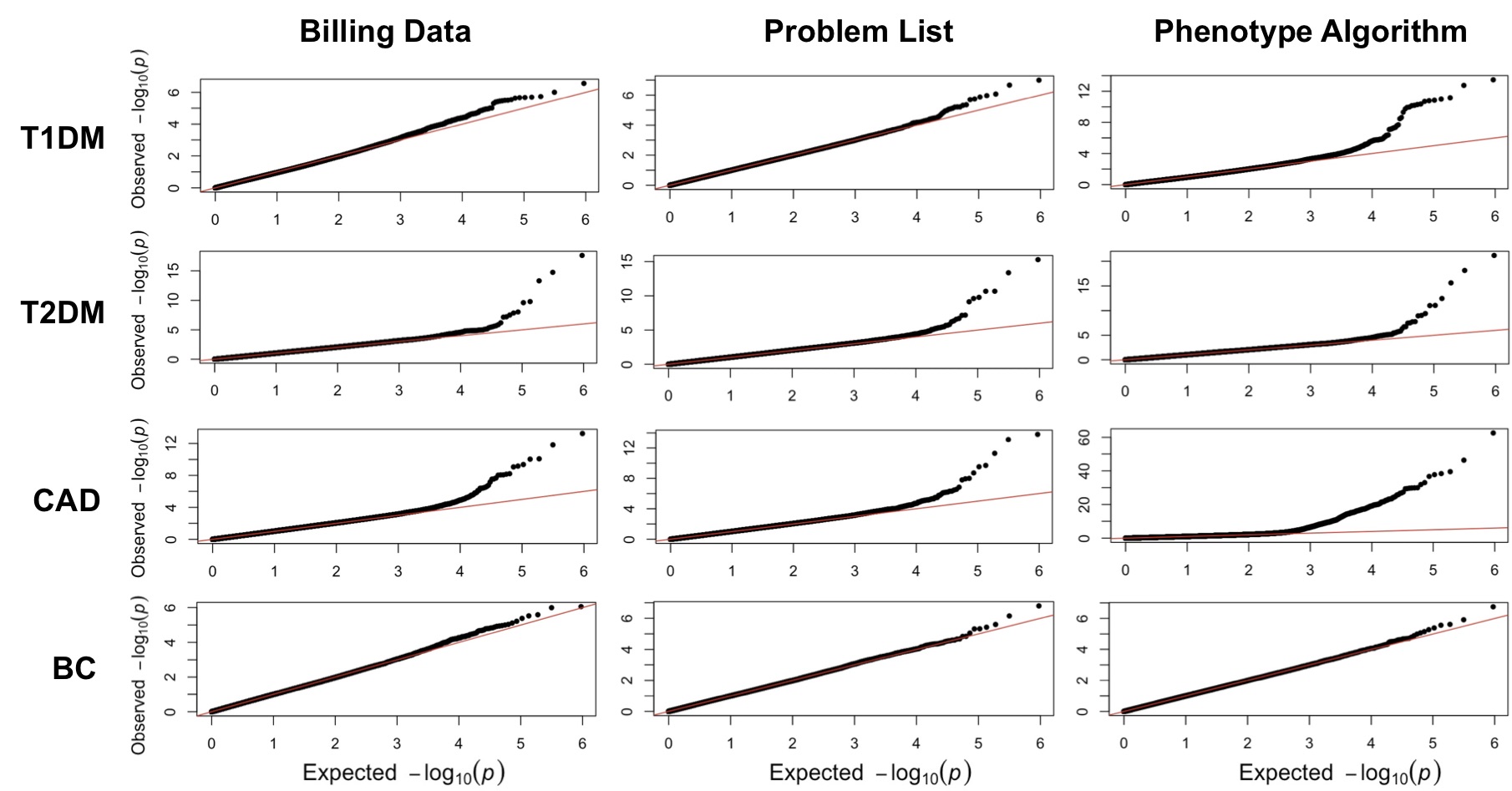
**

**Extended Data Figure1.** QQ plots for logistic regression models in four diseases using three different EHR phenotype extraction methods.

**Extended Data Figure2. The distributions of patients identified by billing code sub-phenotypes**

**
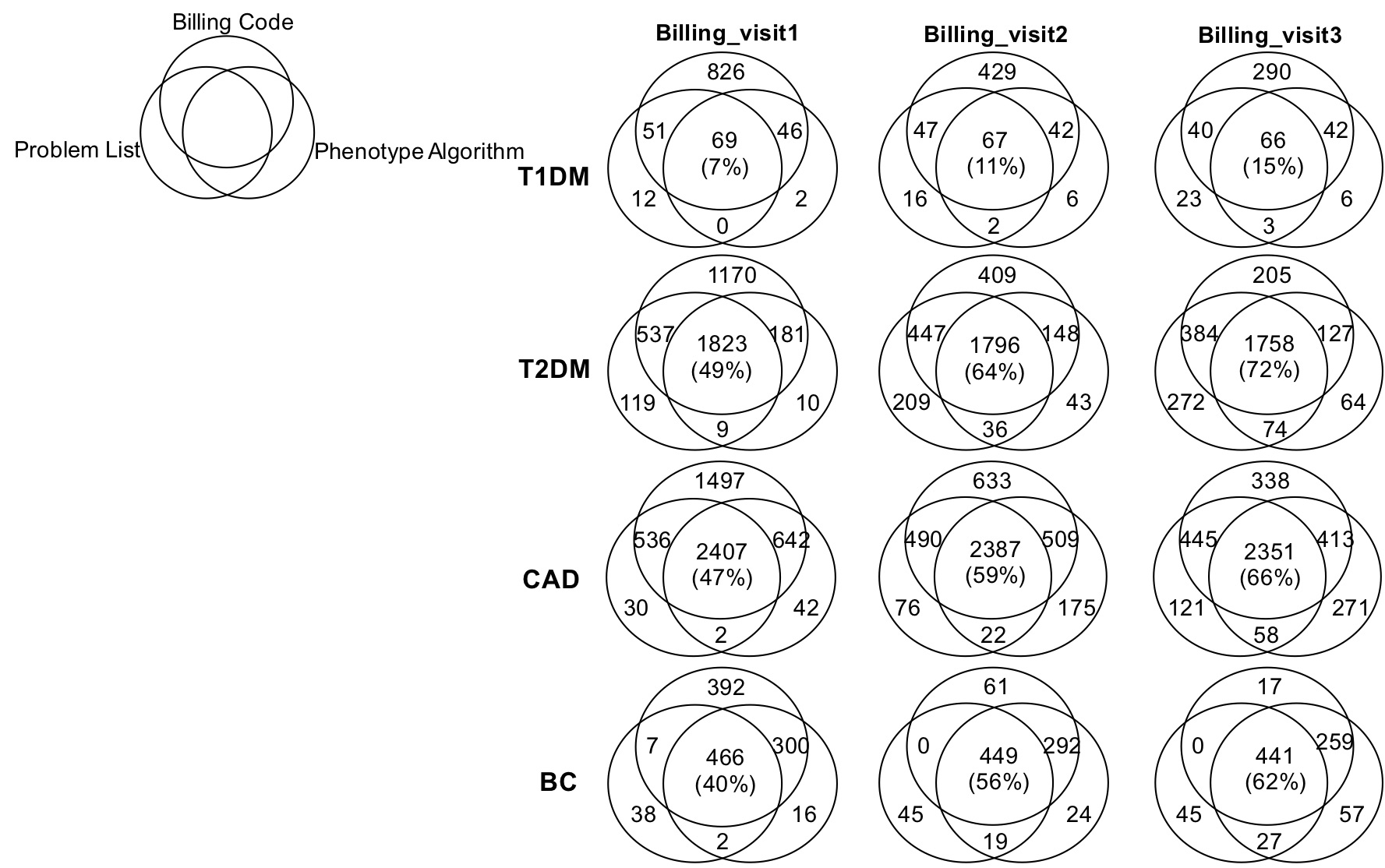
**

**Extended Data Figure2.** The numbers inside the parenthesis are the percentages of patients identified by all the methods accounted for billing data alone patients
